# Exploring the Experience of Healthcare Workers Who Returned to Work After Recovering From COVID-19: A Qualitative Study

**DOI:** 10.3389/fpsyt.2021.753851

**Published:** 2021-11-08

**Authors:** Hui Zhang, Dandan Chen, Ping Zou, Nianqi Cui, Jing Shao, Ruoling Qiu, Xiyi Wang, Man Wu, Yi Zhao

**Affiliations:** ^1^Department of Cardiology, Guizhou Provincial People's Hospital, Guiyang, China; ^2^Zhejiang University School of Medicine, Sir Run Run Shaw Hospital, Hangzhou, China; ^3^Department of Scholar Practitioner Program, School of Nursing, Nipissing University, Toronto, ON, Canada; ^4^Department of Nursing, The Second Affiliated Hospital, Zhejiang University School of Medicine, Hangzhou, China; ^5^Shanghai JiaoTong University, School of Nursing, Shanghai, China; ^6^Department of Nursing, Hubei No. 3 People's Hospital of Jianghan University, Wuhan, China

**Keywords:** healthcare workers, return-to-work, COVID-19, mental illness, stress

## Abstract

**Background:** To date, a large body of literature focuses on the experience of healthcare providers who cared for COVID-19 patients. Qualitative studies exploring the experience of healthcare workers in the workplace after recovering from COVID-19 are limited. This study aimed to describe the experience of healthcare workers who returned to work after recovering from COVID-19.

**Methods:** This study employed a qualitative descriptive approach with a constructionist epistemology. Data were collected through semi-structured in-depth interviews with 20 nurses and physicians, and thematic analysis was used to identify themes from the interview transcripts.

**Results:** Three major themes about the psychological experiences of healthcare workers who had recovered from COVID-19 and returned to work were identified: (1) holding multi-faceted attitudes toward the career (sub-themes: increased professional identity, changing relationships between nurses, patients, and physicians, and drawing new boundaries between work and family), (2) struggling at work (sub-themes: poor interpersonal relationships due to COVID-19 stigma, emotional symptom burden, physical symptom burden, and workplace accommodations), (3) striving to return to normality (sub-themes: deliberate detachment, different forms of social support in the workplace, and long-term care from organizations).

**Conclusions:** The findings have highlighted opportunities and the necessity to promote health for this population. Programs centered around support, care, and stress management should be developed by policymakers and organizations. By doing this, healthcare workers would be better equipped to face ongoing crises as COVID-19 continues.

## Introduction

Coronavirus disease 2019 (COVID-19) was declared as a pandemic and a public health emergency of international concern by the World Health Organization (WHO) in 2020 (1). This disease is causing an unprecedented threat and a global public health crisis. Millions of infected cases and hundreds of thousands of deaths have been reported globally, with a greater threat of fatality for this the elderly and other vulnerable populations (2). Additionally, due to COVID-19, health services were disrupted, so healthcare systems need to change service delivery and prioritize patients with the most urgent needs. To respond to the increased demand, health services were required to make significant changes and respond rapidly to deliver high-quality care to save lives.

In clinical settings, nurses and physicians have longer and more frequent physical contact with COVID-19 patients, which increases the risk of cross-transmission and occupational exposure. According to a recent systematic review, 152,888 healthcare workers were infected by COVID-19 across 195 countries (3). After recovery from COVID-19, many healthcare workers returned to work as they perceived they were able to work and met the return-to-work criteria. Healthcare workers returning to work can help mitigate the depletion of the workforce in hospitals and increase the capacity of wards in the current COVID-19 pandemic (4). It is evident that healthcare workers who returned to work after recovering from COVID-19 could face challenges due to the psychological stress and physical problems caused by this severe disease. This is supported by research indicating that Severe Acute Respiratory Syndrome (SARS) survivors reported poor quality of life, due to many health burdens, such as physical and mental health issues (5).

However, the topic of healthcare workers who were infected with COVID-19 and are returning to work has been paid little attention. Different sources of distress may exist for this specific group when they return to work. First, healthcare workers have expressed certain psychological, physical, and psycho-social challenges when infected with COVID-19. According to a systematic review, the most common physical symptoms among infected healthcare workers were fever, dry cough, anosmia, and myalgia (6). Anxiety and depression were also frequently reported in qualitative and quantitative studies (7, 8). All these influences could lead to psychological issues when this specific population returns to work, as a previous study found that people with physical symptoms and physical problems will have severe psychiatric symptoms after they return to work during the pandemic (9). Second, evidence also shows that disease survivors find they need to face changes in the workplace including accommodations in workloads, productivity loss, or diminished work ability caused by disease (e.g., tasks and hours) (10), when returning to work. All these negative influences may cause harmful effects, including poor work performance, low productivity, poor quality of care, and medical errors for healthcare workers returning to work after recovering from COVID-19.

To date, a large body of literature has focused on the experience of healthcare providers who care for COVID-19 patients. Limited qualitative studies have explored the experience of healthcare workers who returned to work after recovering from COVID-19. We found that the existing evidence relating to healthcare workers returning to work after infection in previous pandemics/outbreaks focused only on the numbers of healthcare workers who returned to work rather than exploring the relevant issues healthcare workers faced while recovering and choosing to return to work (11, 12). The COVID-19 pandemic allows us to answer this unsolved question. Therefore, scholars are calling for more relevant evidence to support healthcare workers who returned to work after recovering from COVID-19 to improve their well-being now and in the future (13).

This study aimed to describe the experiences of healthcare workers who returned to work after recovering from COVID-19. A large number of healthcare workers around the world are getting involved in the COVID-19 pandemic making great contributions, although they face great risks. The number of healthcare workers who returned to work after recovering from COVID-19 is also increasing on a global scale. Therefore, relevant issues and challenges should be investigated to explore their experiences and gain insight for sustaining well-being, psychosocial care, and mental healthcare (14). In-depth analysis and knowledge are needed to understand the stress, problems, and coping strategies of this specifically targeted population. In such cases, healthcare workers will be able to return to normality in the workplace and be better equipped to face ongoing crises as the COVID-19 pandemic continues. Finally, health services would subsequently become more efficient to provide high quality care for more patients in this crisis. Global policymakers could benefit from evidence to inform policy and implementation programs improving health among this population.

## Methods

### Study Design

To better understand the experiences of healthcare workers who returned to work after recovering from COVID-19, this study used a qualitative descriptive approach with a constructionist epistemology to provide comprehensive information about an event (15). This epistemology acknowledges that knowledge is constructed from an individual's perception and experiences, and constructed via speech to understand the world (16). Our study adopted this epistemology as meanings can emerge from the active engagement of the researcher with the participant through a bidirectional understanding of the experience relationship, where language is viewed as implicit in the social production and reproduction of both meaning and experience (17).

### Ethical Considerations

Ethics approval for this study was received from the institutional review board at Hubei No. 3 People's Hospital of Jianghan University (2020-047). This study was conducted in accordance with the declaration of Helsinki.

### Study Participants and Data Collection

Due to the nature of their jobs, nurses and physicians have the longest physical contact with COVID-19 patients and are more likely to be at risk of cross-transmission and occupational exposure. Evidence showed nurses were the largest group of healthcare workers with COVID-19 infection and physicians were the largest group of healthcare workers who had died from the virus (6). We decided to choose both nurses and physicians in our final sample, which was appropriate and representative to explore this target population's experience.

The participants were recruited purposively in a tertiary hospital in Wuhan, in June 2020. We first contacted the hospital's department of nursing and gained information about the nurses infected with COVID-19 who had recovered and returned to work. The inclusion criteria were infected healthcare workers who had recovered from COVID-19 and returned to work for at least 3 days. Additionally, they agreed with participation. Maximum variation sampling was used regarding age, work experience, occupation, days of return to work, and departments. Additionally, snowball sampling was employed for supplement selections. We asked included participants to help recruit other participants, especially physicians. After reaching and identifying participants, they were told about the aims of the study along with the risks and benefits of participation. Researchers explained that participation was anonymous and confidential, and participants were given the chance to decline participation.

Semi-structured in-depth interviews were conducted and the interviews lasted approximately 40–60 min. Informed consent was obtained before each interview. If participants experienced distress during interviews, they could decide on their own to discontinue the interview and the interview was rescheduled for a later time. No participants withdrew from this study. All audio recordings and transcripts were saved on a password-protected computer. In total, 20 nurses and physicians were interviewed for this study because thematic saturation had been reached.

Based on recommendations from a qualitative semi-structured interview guide (18), we conducted an extensive literature review focused on the purpose of the study to forming a conceptual basis for the interview and subsequently, a list of questions as an interview guide was defined. These questions can help researchers gain spontaneous and in-depth answers from participants. Next, we held a meeting with the research team to evaluate the preliminary interview guide by removing ambiguities and leading questions; after this, we invited an external specialist to assess whether the interview guide contents were appropriate and comprehensive for the aims and the subjects of the study. Finally, a qualitative interview guide was formed.

### Data Analysis

The language content of recorded interviews was transcribed verbatim, and each transcript was double-checked for inaccuracies. To assure confidentiality, we used numbers instead of names (eg, physician P1, P2, etc, and nurse N1, N2, etc) and removed identifying information from the transcripts.

Thematic analysis was used to identify themes from the interview transcripts. This data analysis strategy is an approach used to identify and analyze patterns of meaning from data (19). It can help illustrate important themes in the description of a phenomenon. Themes were drawn from the theoretical idea and from the raw data itself. During the thematic analysis process, we first completed a thorough overview of all the data and took initial notes, by reading and rereading the transcripts. Then, by highlighting interesting phrases or sentences, codes were generated to describe their content. Themes were generated by identifying patterns among codes, and we also searched for data that was relevant to each theme. Themes were reviewed to ensure they were useful and accurate representations of the data by splitting, combining, discarding, or creating new themes. Based on a final list of themes, each theme was named and defined. By doing this, these themes give a better understanding of the data. Throughout the analysis process, the research team members wrote memos and held meetings to review findings, identify themes, cross-check initial codes.

## Results

In this study, a total of 20 physicians and nurses participated in interviews, and sociodemographic characteristics are shown in Table 1.

**Table 1:** Characteristics of participants.

	**Age**	**Gender**	**Marital status**	**Work experience (years)**	**Department**	**Education**	**Return to work (days)**	**relapse**
**N 1**	43	Female	Married	24	Cardiology	Bachelor degree	4	2
**N 2**	44	Female	Married	25	Infectious disease	Bachelor degree	87	0
**N 3**	50	Female	Married	28	Emergency room	Bachelor degree	46	0
**N4**	26	Female	Single	3	Thoracic Surgery	College diploma	37	0
**N 5**	38	Female	Married	17	Radiology	Bachelor degree	54	1
**N6**	27	Female	Single	3	Neurology	Bachelor degree	40	0
**N 7**	28	Female	Single	6	Neurology	College diploma	40	1
**N 8**	48	Female	Married	29	Radiology	College diploma	33	0
**P 9**	39	Male	Married	14	Respiratory medicine	Master degree	24	0
**N10**	33	Female	Married	10	Pharmacy Intravenous Admixture Services	College diploma	49	0
**N11**	39	Female	Married	21	Dialysis center	College diploma	12	1
**N12**	29	Female	Married	8	Pain medicine	Bachelor degree	10	0
**N13**	34	Female	Married	11	Neurology	Bachelor degree	18	0
**N14**	35	Female	Married	13	Hematology	Bachelor degree	12	0
**N15**	34	Female	Married	15	Obstetrics and Gynecology	College diploma	15	0
**N16**	37	Female	Married	16	Endocrinology	Bachelor degree	5	2
**P17**	35	Male	Single	12	Radiology	Bachelor degree	40	0
**P18**	38	Male	Married	14	Neurology	Master degree	4	0
**P19**	42	Male	Married	17	Plastic surgery	Master degree	17	0
**N20**	30	Female	Single	10	Hepatobiliary Surgery	College diploma	7	0

Thematic analysis yielded three major themes on the experiences of healthcare workers who had recovered from COVID-19 and returned to work. These themes are (1) holding multi-faceted attitudes toward the career (sub-themes: increased professional identity, changing relationships between nurses, patients, and physicians, and drawing new boundaries between work and family), (2) struggling at work (sub-themes: poor interpersonal relationships due to COVID-19 stigma, emotional symptom burden, physical symptom burden, and workplace accommodations), and (3) striving to return to normality (sub-themes: deliberate detachment, different forms of social support in the workplace, and long-term care from organizations).

### Holding Multi-Faceted Attitudes Toward the Career

#### Increased Professional Identity

After experiencing COVID-19, participants re-evaluated the value of their occupation showing a positive attitude to their work. They felt supported and their occupation was deemed respectful, so they had a high level of professional identity. Many physicians and nurses volunteered and agreed to care for patients with COVID-19 despite the risk of potential infection when they felt it was their duty to save lives. The personal sacrifice of healthcare workers was acknowledged by society and communities, and they were regarded as heroes by the entire society.

N5 “*Due to the events that happened this year, some changes have taken place. For example, the citizens highly praise our career and people around me all honor me as a hero. Actually, this is our job and duty to rush ahead in the front line”*

#### Changing Relationships Between Nurses, Patients, and Physicians

An interesting finding was that participants wanted to improve the relationships between nurses and physicians. Physicians used to hold negative attitudes toward nurses since they viewed the tasks of a nurse to be very simple, such as infusion and dispensing of medicine, so the relationships between physicians and nurses were sometimes strained and their communication was poor. However, physicians infected with COVID-19 believed that the value of nurses should not be overlooked and underestimated. When physicians suffered from COVID-19 as patients, they realized that nurses played a critical role, and they appreciated their nursing colleagues as they helped patients get dressed, provided oral hygiene and skin care, and gave words of encouragement to help patients fight diseases. Meanwhile, nurses thought that they were overworked, underappreciated, and underutilized in the workplace and hoped that physicians would understand, support, and respect them for better interprofessional collaboration.

P17 “*I didn't know the importance of the nursing profession and had no idea about their pains. This close experience let me have a new understanding of the nurse profession. I admire them very much. Actually, you have been closest to the dreadful virus and accompanied with patients for the longest time, which make me very moved. I don't think I can do so well. In my future career, I will definitely respect nurses more and care for them more”*N15 “*I hope the doctors will treat the nurse with more care. The doctors had better consider nurses as colleagues and companions rather than assistants or subordinates”*

Another profound finding was that participants expressed they wanted to provide more patience, sympathy, and empathy, to patients than before to build a positive relationship between patients and healthcare workers. After suffering from COVID-19, healthcare workers had experienced the role of patients. They re-evaluated their duty and responsibility, and they realized they should not only focus on treating disease, but also provide patients with more care, empathy, patience, love, and understanding. Many of them described that they wanted to consider problems from the patients' perspective more in the future, and developed advanced medical skills to deliver high-quality care to patients to mitigate their discomfort.

N6 “*Now I shall count it as a personal favor because it was my first to be in the hospital. After the transposition experience, I think I should treat my patients with more patience. I should consolidate my professionalism so that I can relieve the pain of my patients…In my future long career, I will try my best to help the patients in need, satisfying their basic life needs at the utmost”*

#### Drawing New Boundaries Between Work and Family

As a physician or a nurse, it is difficult for them to maintain their work-family balance in daily life due to heavy workloads, insufficient vacation time, and demanding requirements from clients. After returning to work, some participants have drawn new boundaries between work and family. They re-assessed the values and priorities in their lives and believed that family is the most important thing in the world. Consequently, they felt that they should spend more time with family rather than at work to make changes to their life orientation.

P19 “*In the past, I always insisted on working late. Now I will waste no time to get the job done and go home early. I get to know the importance of my family through this matter. And I truely feel that I used to spend little time with my children, which makes me to return to family and focus of my child.”*

### Struggling at Work

#### Poor Interpersonal Relationships Due to COVID-19 Stigma

Due to high human-to-human transmissibility and the possibility of recurrence, the vast majority of participants reported that they were suffering from COVID-19 stigma including the internalized stigma and externally experienced stigma influencing their social health and interpersonal relationships in the workplace. Although healthcare workers who recovered from COVID-19 had passed several COVID-19 tests before they returned to work, many of them still believed that they were patients and perceived internalized stigma. Participants thought they were still contagious and adopted rigorous approaches such as reducing contacts to reduce the possibility of transmission. This led to poor social health in the workplace. On the other hand, participants experienced discrimination from their colleagues and experienced external COVID-19 stigma. Some participants described experiences when their colleagues deliberately reduced social interactions with them due to the belief that they were still carriers of the COVID-19 virus after recovery. Many participants believed that they were treated unfairly because they heard rumors, and experienced discrimination and misunderstanding in the workplace.

N8 “*While they were eating in the canteen, I, facing the cold wall, was eating in a small corner just by myself. When I worked, I always wore two masks and washed my hands dozens of times a day for fear that I might be a patient and would infect others”*

N3 “*They believed I was a patient and was contagious. This was one of the reasons that caused some special behaviors. For example, they were not willing to let me help them wear personal protective equipment. At the same time, they were not willing to sit in the chair that I had been seated before. Even if they had to sit there, they always used alcohol disinfection before sitting. Don't you think it was funny? Would they be infected by virus through trousers?”*

#### Emotional Symptom Burden

Participants not only needed to take care of patients, but also had to manage their own negative emotions after they returned to work. Participants felt fearful, vulnerable, anxious, and depressed when they returned to the workplace, due to the uncertainty of the consequences of COVID-19, such as unknown complications, pathogenicity, and recurrence. They worried that they could be re-infected and have to face quarantined, which was a horrible memory for them. Some participants who experienced relapse described they were helpless and desperate. They also felt a diminished sense of power and thought they may never have a healthy body because they had to live with the virus forever.

N11 “*I felt desperate at that time, because this disease is unknown and we cannot predict the future. I haven't been reunited with my family members yet…All returned staff need to be checked in time according to the instructions. Something bad happened to me, that is, the result of my test detection is positive”*

#### Physical Symptom Burden

Participants frequently described physical symptom burden after returning to work, such as physical fatigue, sleep difficulties, gasping for air, cough, and difficulties with standing for long periods of time. Some participants believed these physical limitations could affect their work performance. This fear prompted them to want to increase their workload step by step, apply for a limited workload and reduce working hours. The discrepancy between workloads and actual ability to work became difficult to manage for many of the participants in the workplace.

P18 “*It will take some time to let me return to my best physical state completely”*

N20 “*Due to the lack of physical strength, I can just be an assistant or nurse the mild patients rather than critical patients”*

#### Workplace Accommodations

Because of COVID-19, participants found that all working conditions and working standards were changed when they returned to work. Some participants needed to learn how to wear personal protective equipment (PPE) correctly to protect themselves and deliver medical treatment. Some found that they needed to learn updated guidelines, new skills, and new working standards.

N15 “*Back to work, I found that the personal protective equipment may bring difficulty in our working time, such as unclear vision, bad stitching time.”*

N13 “*Currently, there are some changes in the responsibilities and processes of each shift. It was a little chaotic at the beginning and I was afraid that I could not sort it out well.”*

### Striving to Return to Normality in the Workplace

#### Deliberate Detachment

In order to return to normality in the workplace, participants made efforts to detach themselves from the negative emotions and avoid being overly emotionally affected. They understood that they may get out of control, anxious, depressed, and desperate, so deliberate detachment was an effective way to help them remain calm at work and not become overly emotionally affected. For example, some of the participants immersed themselves in patient care and paid little attention to the information about COVID-19 to manage their negative emotions and avoid being emotionally affected by the horrible information around them.

N14 “*I always worry about that if my lung nodules could be cured completely or it would be infected in the future once again. After all, this disease has the possibility of relapse. I force myself to focus on my work instead of thinking about this terrible disease. In this way, time flies very fast”*

#### Different Forms of Social Support in the Workplace

A supportive work environment played a vital role when participants returned to normality in the workplace. Participants expressed that due to multiple support systems in the workplace, they were able to return to normality in the workplace successfully. Support from their supervisor and colleagues helped them to adapt to their work. The various forms of social support included emotional supports from supervisors and colleagues, instrumental support such as knowledge sharing, workplace accommodations, and time to rest. Especially, participants were willing to gain support from other healthcare workers infected with COVID-19. Although support from their colleagues and supervisors helped them adapt to work, support from the infected health worker group made them confident, calm, and relaxed when they return to work. Participants often described that encouragement from infected health workers made them feel like they were not alone and gave them a sense of safety in the workplace.

N5 “*Our department staff are so nice. In the beginning, I didn't dare to go into the lounge and just stood outside when I spoke. However, they always waved to me, saying, why not come in? we don't mind actually Gradually, we began to have breakfast together and they treated me as before. It made me feel warm”*

N13 “*But I preferred to stay with the infected colleagues like me. We were infected at the same time and hospitalized together…we communicated with each other during isolation. I didn't want to make my family members worried about me and other colleagues couldn't understand my feelings so that I didn't want to talk with them at that time. I would rather talk with the infected colleagues who had the same experience as me and we could encourage each other in the workplace”*

#### Long-Term Care From Organizations

After experiencing COVID-19, some participants expressed that the return to normality was a long-term process in the workplace, as they needed to struggle with many difficulties, such as damage of pulmonary functions and relapse. Consequently, their organizations should make plans and programs to provide them with long-term care for psychological health and physical health (e.g., mental health intervention annual health examinations).

N5 “*I hope the hospital can help establish the long-term health records to track recovery*”

P9 “*I think everyone still has traumatic sequelae. If possible, please send someone to talk to us in the future."*

## Discussion

To our knowledge, this is the first study that explores the experiences of healthcare workers who returned to work after recovering from COVID-19 in China. The findings provide a holistic view of the experiences of medical staff who returned to work after recovering from COVID-19.

After experience infection and returning to work, healthcare workers re-evaluated their occupation and held multi-faceted attitudes toward their careers. Some healthcare workers believed that their professional identity was enhanced. Professional identity is important to the core or essential aspects of professional value. In this public health crisis, dedication and commitment derive occupational calling. After hearing the call for duty, many healthcare workers devoted themselves to the pandemic by applying their professional knowledge and their highest level of skills to treat patients with COVID-19 (20). Although healthcare workers needed to care for and treat COVID-19 patients in demanding work environments, they felt that their work was acknowledged, respected, and valued. Their occupational skills and personal sacrifices were highly valued and praised by COVID-19 patients and society.

Other participants wanted to build positive relationships between nurses, physicians, and patients after returning to work. Physician-nurse relationships have always been a crucial issue, and are characterized by a lasting pattern of physician dominance and nurse deference, leading to increasing conflict (21). Sex roles, education, and social-economic status, all contribute to fundamental disparities in knowledge and power between nurses and physicians (22). Therefore, the nursing role has gradually become undervalued in the workplace. However, some physicians mentioned that they had previously misunderstood the value of nurses, and the importance of nurses should not be overlooked and underestimated. After being a patient, they realized that nursing care is important to patients' physical health and psychological health. The positive relationship between nurse and physician is vital to a supportive work environment that enhances collaboration and improves the quality of patient care (23).

Moreover, many participants were willing to improve the relationships between patients and healthcare workers. In clinical practice, healthcare workers are more likely to employ scientific knowledge, expertise in diagnosis, and excellent technique to treat disease. However, many “soft skills” are also important when providing person-centered care and often times overlooked (24). These soft skills are sympathy, empathy, and compassion. After experiencing the role of a patient, healthcare workers believed sympathy, empathy, and compassion were vital in healthcare. Evidence has indicated that emotional resonance can help improve patient-reported outcomes, quality of care, and patient satisfaction, due to a deeper understanding of the person and their individualized suffering (25).

Because of workloads, work shifts, and long working hours, healthcare workers suffer from work-family conflict (26). Many of them used to sacrifice parts of their family life to take on the responsibility of caring for their patients (27). However, after infection with COVID-19, these healthcare workers considered drawing new boundaries between work and family when they returned to work. This means that they want to spend the amount of time with their family members. It is difficult for healthcare workers to balance professional and personal obligations, and a poor balance of work and family life has a negative effect on retaining healthcare workers (28). Previous evidence suggested that flexible work hours, supportive relationships with spouses and partners, parents, and members of the community, as well as training programs for balancing work with personal roles can help maintain the balance between work and family life (27). Hospital management should make an effort to enable healthcare workers to build successful, durable, and satisfying careers.

We found that participants were struggling in many ways when they returned to work. Participants suffered from COVID-19 stigma in the workplace after recovering from COVID-19, resulting in poor interpersonal relationships. We found this aligns with existing literature (29). Great fear could be triggered, due to the high risk of COVID-19 such as person-to-person transmission chains, relatively long incubation period, and some asymptomatic cases of COVID-19 (30). Because the virus is highly contagious, fatal, and uncontrollable, some participants had doubts that their bodies were still carrying the virus after returning to work. Moreover, some participants reported that their co-workers and supervisors had reacted negatively toward them, and discriminatory words were said to them. The occurrence of transmission led people to adopt instinctual behavior as people naturally avoided and isolated individuals who were infected with COVID-19 (31). This kind of behavior may reduce risk of exposure, but it can stigmatize individuals who have recovered from the disease. The perceptions of stigma relating to COVID-19 can have a detrimental impact on interpersonal relationships, resulting in poor collaboration in the workplace. Therefore, it is necessary to raise awareness that increased communication about COVID-19 is crucial to avoid discrimination and stigma in a work environment. The hospital management should make an active effort to scientifically destigmatize COVID-19 by providing sufficient health education targeting the workforce, maintaining the privacy and confidentiality of infected healthcare workers, and reporting the unfair treatment experience of people who suffer from stigma due to COVID-19 (32).

Our results suggested that one of the difficulties was that many participants were emotionally overwhelmed and expressed a wide range of negative emotions including fear, desperation, anxiety, depression, powerlessness, and frustration after returning to work. Previous studies indicated that people who had been affected by pandemics as survivors, caregivers, or health professionals working with infected patients, were more likely to suffer negative emotions (33, 34). This was caused by the characteristics of COVID-19 (e.g., negative outcomes, relapse, complications, and contagion). This means that participants had less knowledge about relapse and risk of contagion, which contributed to a sense of helplessness and uncertainty. More importantly, healthcare workers were in constant fear of becoming re-infected due to unintentional occupational exposure. Psychological distress among healthcare workers is detrimental, as it has a negative impact on their work performance, quality of care, quality of life, and wellbeing. Hospital management should adopt a wide range of consultant services and support to meet the unique needs of this population and minimize the devastating impact of COVID-19 on wellbeing in the workplace (35).

Experiencing COVID-19 not only affects healthcare workers mentally, but also physically, after they returned to work. The findings in our study align with other studies that sleep disorders, muscle weakness, fatigue, and diminished physical capability were reported among these individuals when they returned to work. Supervisors should gradually increase the workload step by step for healthcare workers who return to work. Moreover, particular attention needs to be paid to gender considerations when designing and implementing plans to help healthcare workers adapt to work. A previous study suggested that women were more likely to suffer physical decline or fatigue, post-activity polypnoea, and alopecia than men (36). Moreover, Huang et al. found that patients with severe disease are the major target population for interventions of long-term recovery because these patients have increasingly impaired pulmonary diffusion capacities and abnormal chest imaging manifestations during their hospital stay (37). Therefore, hospital management should implement long-term follow-up visits for infected healthcare workers to monitor their health condition.

Regaining normality in the workplace was of paramount importance to the participants. Participants made efforts to rebuild normality, and several resources and strategies were crucial. Some participants made active choices by detaching themselves from their concerns of health and avoided exposing themselves to negative media coverage. This kind of emotion-focused coping puts emphasis on managing internal emotive responses to stressors, which can divert their attention from negative thoughts and escape unpleasant realities (38). After returning to work, healthcare workers found that working conditions had changed greatly. Most participants were making efforts to adapt to new rules, sanitation procedures, standard operating procedures, updated guidelines, and risk management policy. In order to accommodate a new working environment, support and information from co-workers and supervisors are vital. By seeking social support, participants can minimize and manage stressors (39). It is suggested that good organizational leadership, tailored support, personal recommendations, and modified work arrangements from a supportive workplace are implemented. Moreover, interpersonal support from the infected health worker group led to a sense of togetherness with others who had similar experiences, which helped participants cope with the uncertainty of the future. Hospital management should develop programs to allow this particular group to have opportunities to discuss and share their own experiences with healthcare workers who have had similar experiences. When participants were striving to regain normality, they also expressed that long-term care from organizations for mental health and physical health were needed. Previous evidence suggested that pandemic survivors were suffering from long-lasting psychiatric symptoms morbidity (e.g., chronic PTSD) and physical symptoms (e.g., lung abnormalities) (11, 40). Hospitals should provide long-term care to monitor and facilitate survivors' long-term physical and mental recovery.

Compared with previous studies, the contribution of this study is two-fold. First, studies on previous pandemics ignored the experience of healthcare workers who returned to work after infection and placed an emphasis on the number of healthcare workers who returned to work after infection. However, this study provided profound results about the difficulties and problems of this group during the process of returning to work. Second, most research focused on the experience of healthcare workers who took care of patients infected with COVID-19 or infected with COVID-19. However, the vital experience of healthcare workers who returned to work after infection was overlooked, and this special group has received little attention until now. Only one qualitative study has been conducted on this topic, but the sample of the study included only nurses rather than both nurses and physicians (41). Our study investigated both nurses and physicians to provide comprehensive evidence on this topic. Additionally, various cultures can result in different experiences and opinions. This previous qualitative study was conducted in Iran, whereas our study was carried out in China, presenting a unique perspective on this issue. Lastly, our study added more details about the valuable resources needed to regain normality and the different attitudes toward the participants' careers. Global policymakers could benefit from combining these findings with existing evidence to inform policy and implementation programs improving health among this population.

### Limitations

This study has some limitations. First, participants may not be representative of all healthcare workers who returned to work after recovering from COVID-19. However, by using maximum variation sampling, participants were selected to represent a wide range of individuals. Second, the majority of health workers were nurses in wards, and nurses were the most commonly infected occupation (6), so there were only four physicians included in this study. However, the purpose of this study is not to compare experiences between nurses and physicians, and the physicians provided full and rich data to ensure data saturation (42).

## Conclusion

The current study provides a comprehensive understanding of the experiences of healthcare workers in the workplace after recovering from COVID-19. Across these conceptual themes, there was an overarching focus on helping to improve well-being in this targeted population. Moreover, clear and evident information about COVID-19 should be offered by experts, supervisors, and managers to minimize the impact of rumors and stigma. Programs about support, care, and stress management should be developed by policy makers and organizations. By doing this, healthcare workers would be better equipped to face ongoing crisis as COVID-19 continues.

## Data Availability Statement

The original contributions presented in the study are included in the article/supplementary material, further inquiries can be directed to the corresponding author/s.

## Ethics Statement

The studies involving human participants were reviewed and approved by the Institutional Review Board at Hubei No. 3 People's Hospital of Jianghan University. Written informed consent for participation was not required for this study in accordance with the national legislation and the institutional requirements.

## Author Contributions

HZ and YZ conceptualized the study. HZ, DC, and YZ contributed to the data collection and transcribing. HZ, YZ, and DC drafted the manuscript. PZ, NC, JS, XW, RQ, and MW contributed to review and edit the manuscript. All authors contributed to coding, analysis, and the article and approved the submitted version.

## Funding

This work was supported by Health Commission of Hubei Province Scientific Research Project (WJ2021M204) and the Science Technology Platform and Talent Team Plan Projects in Guizhou Province [Grant 2017(5405)].

## Conflict of Interest

The authors declare that the research was conducted in the absence of any commercial or financial relationships that could be construed as a potential conflict of interest.

## Publisher's Note

All claims expressed in this article are solely those of the authors and do not necessarily represent those of their affiliated organizations, or those of the publisher, the editors and the reviewers. Any product that may be evaluated in this article, or claim that may be made by its manufacturer, is not guaranteed or endorsed by the publisher.
